# Non-destructive estimation of field maize biomass using terrestrial lidar: an evaluation from plot level to individual leaf level

**DOI:** 10.1186/s13007-020-00613-5

**Published:** 2020-05-13

**Authors:** Shichao Jin, Yanjun Su, Shilin Song, Kexin Xu, Tianyu Hu, Qiuli Yang, Fangfang Wu, Guangcai Xu, Qin Ma, Hongcan Guan, Shuxin Pang, Yumei Li, Qinghua Guo

**Affiliations:** 1 State Key Laboratory of Vegetation and Environmental Change, Institute of Botany, Chinese Academy of Sciences, Beijing, 100093 China; 2 University of Chinese Academy of Sciences, No. 19A Yuquan Road, Beijing, 100049 China

**Keywords:** Biomass, Phenotype, Machine learning, Terrestrial lidar, Precision agriculture

## Abstract

**Background:**

Precision agriculture is an emerging research field that relies on monitoring and managing field variability in phenotypic traits. An important phenotypic trait is biomass, a comprehensive indicator that can reflect crop yields. However, non-destructive biomass estimation at fine levels is unknown and challenging due to the lack of accurate and high-throughput phenotypic data and algorithms.

**Results:**

In this study, we evaluated the capability of terrestrial light detection and ranging (lidar) data in estimating field maize biomass at the plot, individual plant, leaf group, and individual organ (i.e., individual leaf or stem) levels. The terrestrial lidar data of 59 maize plots with more than 1000 maize plants were collected and used to calculate phenotypes through a deep learning-based pipeline, which were then used to predict maize biomass through simple regression (SR), stepwise multiple regression (SMR), artificial neural network (ANN), and random forest (RF). The results showed that terrestrial lidar data were useful for estimating maize biomass at all levels (at each level, R^2^ was greater than 0.80), and biomass estimation at leaf group level was the most precise (R^2^ = 0.97, RMSE = 2.22 g) among all four levels. All four regression techniques performed similarly at all levels. However, considering the transferability and interpretability of the model itself, SR is the suggested method for estimating maize biomass from terrestrial lidar-derived phenotypes. Moreover, height-related variables showed to be the most important and robust variables for predicting maize biomass from terrestrial lidar at all levels, and some two-dimensional variables (e.g., leaf area) and three-dimensional variables (e.g., volume) showed great potential as well.

**Conclusion:**

We believe that this study is a unique effort on evaluating the capability of terrestrial lidar on estimating maize biomass at difference levels, and can provide a useful resource for the selection of the phenotypes and models required to estimate maize biomass in precision agriculture practices.

## Background

The world population is estimated to surpass 9 billion by the year 2050, creating an unprecedented pressure on food security and sustainable development of human societies [1, 2]. Precision agriculture is an emerging research field for improving food productivity, which requires accurate and high-throughput screening of phenotypic traits [3]. Among them, biomass is a comprehensive indicator of crop yield, which is critical for improving crop breeding and mitigating food security challenges [4–6]. Maize is one of the three major crops supplying more than half of the cereal foods around the world. Therefore, studying the biomass estimation of maize is of great importance for the advancement of precision agriculture and food security.

Traditional biomass estimation methods mainly rely on field measurements and destructive sampling [7], which are labor-intensive, time-consuming, and unrepeatable. With the development of remote sensing, biomass estimation has entered a repeatable and high-throughput era. For example, Osborne et al. [8] estimated in-season biomass of corn using field hyperspectral data. Wang et al. [5] estimated maize biomass at plot level using airborne hyperspectral data. These methods are easy to be implemented by building regression models between field-measured biomass and remotely-sensed vegetation indexes (e.g., normalized difference vegetation index, NDVI) [9]. However, biomass estimation from vegetation index-based methods usually suffers from saturation effects at high biomass value [6, 10] and is sensitive to light conditions, background reflectance (e.g., soil) [11], and plant structure [6, 12].

Light detection and ranging (lidar) is an active remote sensing technology, which can acquire highly accurate three-dimensional (3D) data of a target by recording the distance of the sensor to the target according to the flight time of the pulsed laser beam [13, 14]. Due to its strong penetration ability and insensitivity to light conditions, lidar has been shown to have better accuracy than other remote sensing techniques in estimating biomass [15–17]. Terrestrial lidar is a ground-based lidar technology and is the main tool for field lidar data acquisition at small scales due to its portability and highly accurate data quality [13, 18]. However, current studies using terrestrial lidar mainly focus on forests [19, 20]. For crop biomass estimation, there are a few studies. For example, Wang et al. [5], Eitel et al. [21], and Tilly et al. [22] demonstrated that lidar-derived height variables were good proxies for estimating biomass of maize, wheat, and paddy rice. Due to height variation is often unnoticeable among breeding lines, 3D variables derived from lidar have also been investigated to estimate crop biomass. For example, Eitel et al. [11] and Walter et al. [23] estimated wheat biomass with terrestrial lidar-derived vegetation volume. Jimenez-Berni et al. [24] estimated wheat aboveground biomass with 3D profile index (3DPI) and 3D voxel index derived from terrestrial lidar data. However, these studies all focus on plot level, and do not allow to derive precise information at the individual plant or even finer level, which is needed to assist breeding and meet the requirements of precision agriculture. Therefore, the feasibility of biomass estimation using terrestrial lidar at fine levels (e.g., individual plant level, leaf group level, and individual organ level) is unknown.

The main challenge in the use of lidar for biomass estimation is how to extract phenotypic traits accurately at different levels, especially at the individual plant level and even finer levels. The prerequisites of phenotypic traits from lidar data at fine levels are the ability of automatically segmenting individual plants, stems and leaves, which has been long-standing challenges. Recent algorithmic advancements that make use of deep learning have solved these bottlenecks [25, 26], making it feasible to estimate biomass. However, how to select phenotypic traits and models for biomass estimation at different fine levels is still unclear.

In this study, we aimed to explore which regression models and phenotypic traits derived from lidar data are best for estimating maize biomass (not biomass density) at four levels, including plot, individual plant, leaf group, and individual organ (i.e., individual leaf or stem) levels. We believe that this research can guide future biomass estimation efforts from terrestrial lidar data at fine levels, which may further contribute to yield estimation, precision agriculture, and food security.

## Study area and data

### Study area

**Fig. 1:**
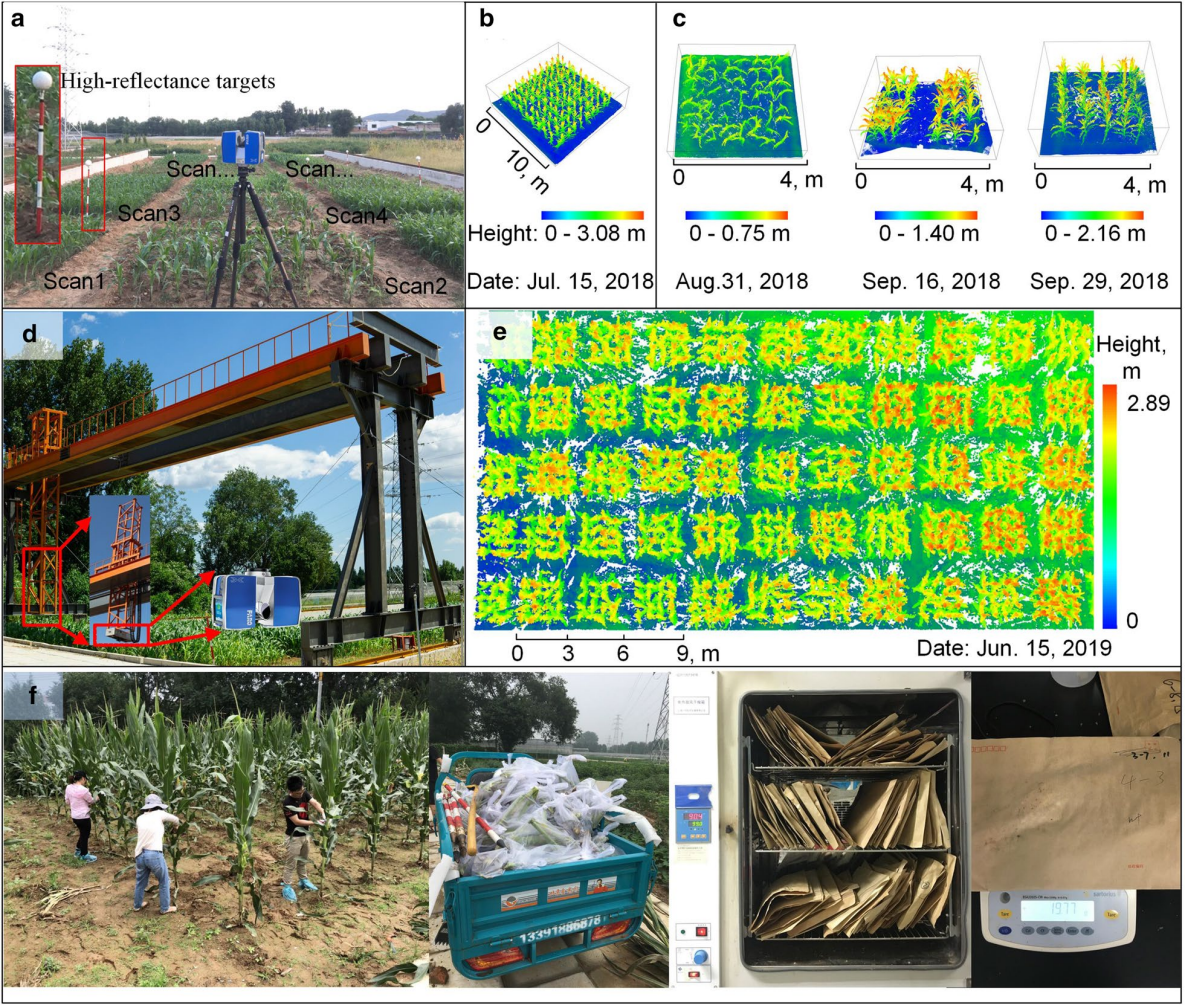
The study area and data collection. **a** Data collection in 2018 using a terrestrial lidar mounted on a tripod. **b** TLS data example collected on July 15, 2018. **c** TLS data example collected on August 31, 2018, September 16, 2018, and September 29, 2018. **d** Data collection in 2019 using terrestrial lidar integrated on a high-throughput phenotyping platform. **e** Terrestrial lidar data example at plot level. **f** Field sampling and biomass measurement at plot, individual plant, leaf group, stem, and individual leaf levels

The study was conducted at the breeding base of the Institute of Botany, Chinese Academy of Sciences (39° 59′ 10″ N, 116°1 2′ 21″ E), in Beijing, China (Fig. 1a). The area of the maize field from which we took the measurements is about 600 m^2^. The mean annual precipitation at the site is 575.16 mm and the mean annual temperature is 12.78 °C. We planted 59 maize plots with the same variety (*Zea mays* L.) in three different periods. In April 2018, only 1 plot was planted, which was used to collect samples for biomass estimation at the individual leaf level. The plot size was 10 m × 10 m, and the planting interval was 1 m in both intra-row and inter-row directions. In August 2018, 3 plots were planted, which were used for collecting samples for biomass estimation at the leaf group, stem, and individual leaf levels. The plot size was 4 m × 4 m, and the planting interval was 0.5 m in both the intra-row and inter-row directions. In April 2019, 55 plots were planted, which were used for collecting samples for biomass estimation at the plot level. The plot size was 3 m × 3 m, and the planting interval was 0.6 m in both the intra-row and inter-row directions (Table 1).

**Table 1:** Description of lidar data and field-measured biomass collected in this study

Planting date	Number of plots	Plot size	Planting interval, m	Number of maize individuals	Lidar data acquisition time	Field biomass acquisition time	Data use
Apr. 10, 2018	1 plot	10 m × 10 m	1	100	Jul. 15, 2018	Jul. 15, 2018	Individual leaf biomass estimation
Aug. 3, 2018	3 plots	4 m × 4 m	0.5	138	Aug. 31, 2018, Sep. 16, 2018, and Sep. 29, 2018	Aug. 31, 2018, Sep. 16, 2018, and Sep. 29, 2018	Leaf group, stem, and individual plant biomass estimation
Apr. 1, 2019	55 plots	3 m × 3 m	0.6	858	Jul. 30, 2019	Jul. 30, 2019	Plot biomass estimation

### Destructive biomass measurements

To collect ground truth biomass at the individual leaf level, we manually sampled all individual leaf samples within the 10 m $\times$ 10 m plot on July 15, 2018 (Fig. 1b, f). These individual leaf samples were first dried at 105 °C for 2 h, and then dried at 65 °C until the sample weight was constant when measured with an electronic scale with an accuracy of 0.01 g. To collect ground truth biomass at the stem, leaf group, and individual levels, we manually sampled 138 individual samples from three different plots at three growth stages, that is August 31, 2018, September 16, 2018, and September 29, 2018. We manually separated the stem from the group of leaves of the three selected plots in the field (Fig. 1c), and weighted them using the abovementioned method in the laboratory. The ground truth biomass at the individual plant level was measured by summing the stem and leaf group biomass of an individual plant.

By contrast, it is hard to dry the plot biomass in the laboratory because there were 858 individual plants. Biomass at the plot level was indirectly measured by converting fresh weight to dry weight according to established relationships built from randomly selected samples [27]. Specifically, all individual maize plants within each plot were first harvested and weighted fresh in the field using an electronic scale with an accuracy of 1 g. After that, we randomly sampled three fresh individual plants of each plot, which were weighted dry at 105 °C for 2 h, and then dried at 65 °C until their weights were constant. According to the samples’ fresh weight and corresponding dry weight, a conversion factor between dry and fresh weighs can be derived, which was further used to calculate the dry weight of each plot.

### Lidar data collection

In this study, lidar data were collected in the morning of the same day as field measurements to reduce the influence of wind. A FARO Focus^3D^ X 330 HDR terrestrial laser scanner was used. The sensor size is 240 mm $\times$ 200 mm $\times$ 100 mm and weighs 5.2 kg. The pulse rate is 244 kHz, and the ranging error is approximately 2 mm (see full details in Table 2). However, the scanning mode and carrying platform were different in 2018 and 2019.

**Table 2:** The specifications of the lidar scanner used in this study

FARO Focus^3D^ X 330 HDR	Specification
Laser wavelength, nm	1550
Laser beam divergence, mrad	0.19
Pulse rate, kHz	244
Maximum scanning rate, Hz	97
Distance accuracy	2 mm @10 m @ 90% reflectance
Field of view, °	Horizontal: 360°; Vertical: 300°
Angular resolution, °	Horizontal: 0.009°; Vertical: 0.009°
Detection range, m	0.6–130 m indoor or outdoor with upright incidence to a 90% reflective surface
Scanner weight, kg	5.2
Dimensions, mm	240 × 200 × 100
Beam diameter at exit, mm	2.25
Laser class	Laser class 1

In 2018, the sensor was mounted on a tripod at the height approximate to the highest canopy height (Fig. 1a). High-reflectance targets were installed randomly within the field with an average distance of about 10 m. Scanning sites were set at each corner of the plots. The operating mode of the sensor was set as “*Outdoor within 10* *m Scanning Profile*” without color information, which is suitable for fixed platforms and can acquire detail information with high efficiency within a short distance (< 10 m). For the maize planted in April 2018, the terrestrial lidar data were acquired for the 10 m $\times$ 10 m plot (Fig. 1b). These data were used to extract phenotypic traits for the estimation of biomass at the individual leaf level. For the maize planted in August 2018, the terrestrial lidar data were collected at three different sites (Fig. 1c). Some plants in the field were removed to facilitate the set-up of the instrument and obtain high-precision data during the scanning on September 16, 2018 and September 29, 2018. These data were used to extract the phenotypic traits for biomass estimation at stem, leaf group, and individual plant levels.

In 2019, lidar data were collected at mature stage in a more efficient way because of the large planted area by mounting lidar sensor on a high-throughput field crop phenotyping system, named Crop3D (Fig. 1d). The system was placed on tracks and moved in x, y, and z directions to cover the field. The lengths of the tracks were 30 m in the north–south (NS) direction, 20 m in the east–west (EW) direction, and 4 m in the vertical direction. The sensor was the same as the one used in 2018 but operated in “*Helical Mode*”. This mode uses the scanner movement information (i.e., scanner speed and direction) to register lidar points from mobile lidar systems, enabling a higher data collection speed. The sensor was mounted at the height of 0.5 m above the highest canopy. The system moved in EW direction at a speed of 0.05 m/s to collect data along a row of plants. Then it moved forward at a distance of 3 m in the NS direction to another row to collect new data. These data were used to extract phenotypic traits for biomass estimation at plot level (Fig. 1e).

## Methods

### Terrestrial lidar data preprocessing

Terrestrial lidar data preprocessing consisted of five procedures: registration, clipping, denoising, filtering, and normalization. First, the data collected in 2018 using a terrestrial lidar mounted on a tripod were registered based on high-reflectance targets; we used the FARO SCENE 6.0 software to get the complete point cloud data of the field. The data collected in 2019 using the Crop3D platform were registered using the system recorded relative location. The registration accuracies for both systems were higher than 2 mm. The data of the target area were then clipped using the Green Valley International^®^ LiDAR360 software. Next, the clipped points were denoised using a statistical outlier removal method included in the LiDAR360 software. The denoised points were filtered into vegetation points and ground points through a local minimum filtering algorithm integrated in the LiDAR360 software. Specifically, this algorithm first uses a moving window to find local minima in height, and interpolates a digital terrain model (DTM) from the local minima. Points above the DTM within a user-defined height threshold are classified as ground points and the other points are classified as vegetation points. In this study, the size of moving windows was set as 0.2 m and the height threshold was set as 0.1 m. Finally, the filtered lidar points were normalized by subtracting the height of each point with the height of its nearest ground point in the horizontal direction. Phenotypic traits were extracted from the normalized lidar point cloud and matched with field measurements.

### Phenotypic trait extraction

In this study, lidar data collected at different levels were used to extract various phenotypic traits (Table 3), including 1D traits (e.g., height), 2D traits (e.g., canopy cover), and 3D traits (e.g., volume) (Table 3). The types and extraction methods of phenotypic traits at different levels are introduced as follows.

**Table 3:** Phenotypic traits extracted at different levels and their statistical information

Levels	Number of samples	Phenotypic traits	Statistics		
			Max	Mean	Min
Plot level	55 plots	H_max_, m	2.894	2.463	1.886
		H_mean_, m	1.314	0.999	0.642
		Height quantiles (H99, H98,…, H80), m	2.463	1.899	1.229
		Canopy cover	0.628	0.320	0.132
		PLA	6.654	4.571	2.249
		Plant area index/PAI	4.174	1.683	0.516
		3DPI	1.717	1.715	1.712
		Volume, m^3^	0.201	0.103	0.010
Individual plant level	138 individual plants	Height, m	2.135	1.089	0.266
		Crown size, m	1.322	0.887	0.178
		CHR	1.562	0.944	0.439
		Canopy cover	0.309	0.173	0.076
		Projected leaf area/PLA, m^2^	0.164	0.076	0.005
		PAI	2.688	0.961	0.301
		3DPI	1.718	1.716	1.710
		Volume, 10^−4^ m^3^	11.521	4.457	0.286
Leaf group level	138 leaf groups	Leaf height, m	2.135	1.077	0.263
		Crown size, m	1.269	0.882	0.179
		CHR	1.731	0.943	0.439
		Canopy cover	0.308	0.171	0.074
		PLA	0.162	0.075	0.005
		PAI	2.396	0.878	0.303
		3DPI	1.718	1.716	1.710
		Volume,10^−4^ m^3^	10.409	4.039	0.269
Organ level	138 stems	Stem height, m	1.536	0.563	0.080
		Stem diameter, cm	3.174	1.898	0.797
		PAI	30.266	10.716	1.901
		3DPI	1.717	1.712	1.695
		Volume, 10^−4^ m^3^	10.049	3.928	0.085
	59 individual leaf samples	Leaf length	1.022	0.701	0.320
		Leaf width	0.200	0.102	0.039
		Leaf area	0.133	0.060	0.012
		PLL	0.852	0.463	0.092
		PLA	0.063	0.029	0.002
		PAI	6.137	1.901	0.290
		3DPI	1.714	1.707	1.669
		Volume, 10^−4^ m^3^	7.900	2.729	0.418

#### Phenotypic trait extraction at plot level

**Fig. 2:**
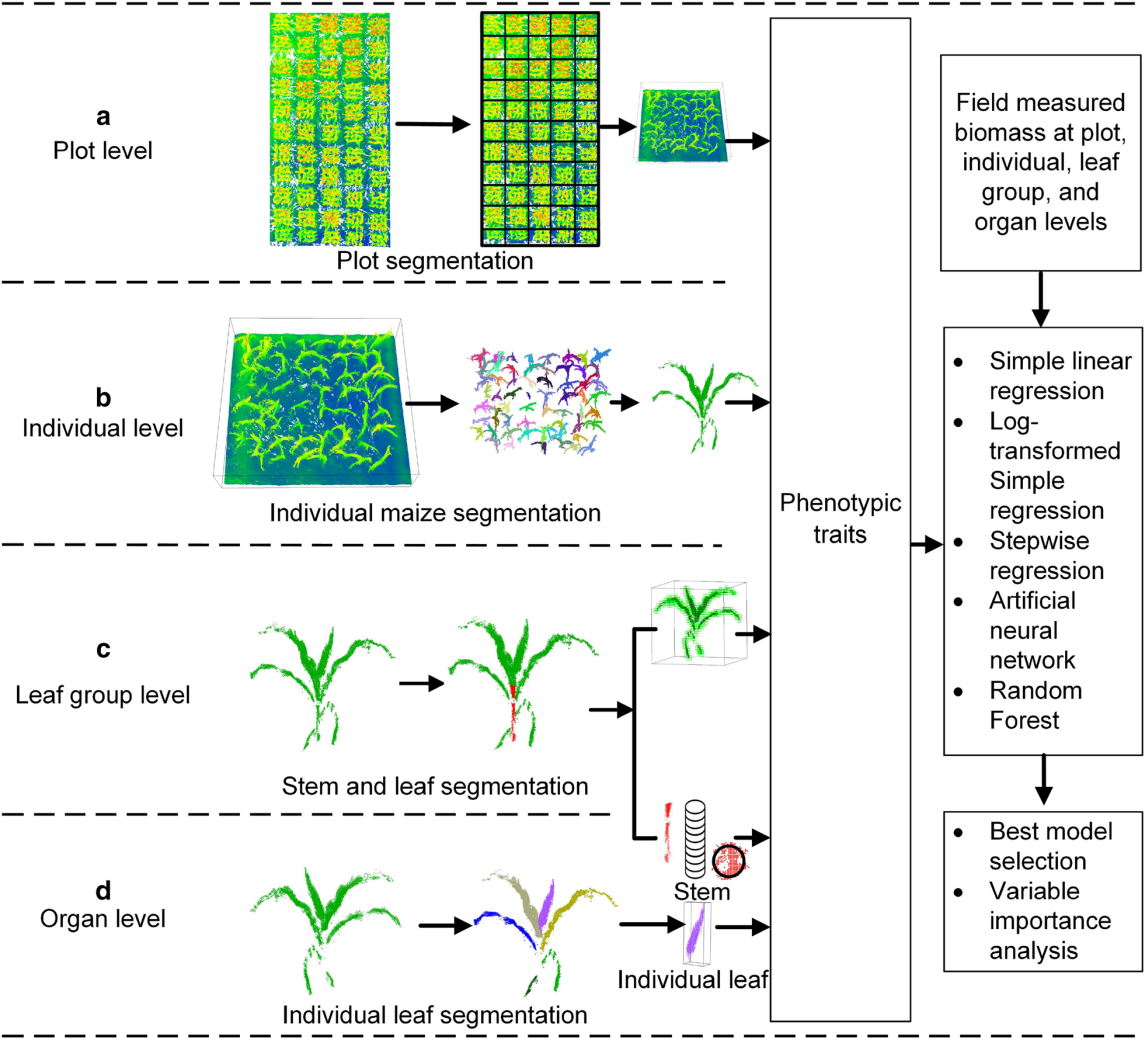
Workflow of the aboveground biomass estimation and selection of the best model and phenotypic trait at different levels. **a** Phenotypic trait extraction and biomass estimation at plot level. **b** Individual maize segmentation, phenotypic trait extraction, and biomass estimation at individual plant level. **c** Stem-leaf segmentation from individual plant, phenotypic trait extraction, and biomass estimation at leaf group level. **d** Individual leaf segmentation, phenotypic trait extraction, and biomass estimation at organ levels, including stem level and individual leaf level

At plot level (Figs. 1e, 2a), data were clipped into 55 individual plots. At each plot, we extracted 27 phenotypic traits (Table 3), including maximum height (H_max_), mean height (H_mean_), height quantiles (from 99% quantile height/H99 to 80% quantile height/H80 with an interval of 1%), canopy cover, projected leaf area (PLA), plant area index (PAI), 3DPI, and volume. H_max_ is defined as the maximum z value of all normalized points. H_mean_ is the mean value of all normalized points. Canopy cover is the proportion of canopy per unit area. PLA is the absolute projected leaf area. In this study, vegetation points were projected into the X–Y plane. The projected points were rasterized with a resolution of 1.5 times of the average point distance by referring to [28]. Then, the canopy cover and PLA were calculated as:

1 $$Canopy \, cover = \frac{{n_{l} }}{{n_{p} }}$$

2 $$PLA = \Delta {\text{x}} \times \Delta {\text{y}} \times \frac{{n_{l} }}{{n_{p} }}$$

where $n_{l} \;{\text{and}}\; n_{p}$ are the number of pixels containing points and the number of all pixels, respectively; $\Delta {\text{x}}\;\text{and}\; \Delta {\text{y}}$ are the length of the target bounding box of the projected points in x and y directions.

PAD, PAI, 3DPI, and volume were calculated using a voxel-based method [29, 30]. All the 3D points were voxelized with a voxel size of 1.5 times of the average point distance [28]. In each horizontal layer ($i$) with a height ($\Delta H$) equal to the voxel size, PAD was computed using Eq. 3.

3 $${\text{PAD}} = \frac{{cos\theta_{c} }}{{G\left( {\theta_{c} } \right)}} \times \frac{1}{\Delta H} \times \mathop \sum \limits_{{i = m_{h} }}^{{i = m_{h} + \Delta H}} \frac{{n_{l} \left( i \right)}}{{n_{p} \left( i \right)}}$$

where $\theta_{c}$ is the laser incident angle; $G\left( {\theta_{c} } \right)$ is the extinction coefficient; *i* is a given vertical layer; $m_{h}$ and $m_{h} + \Delta H$ are the bottom and upper voxel vertical coordinates of layer *i*; $n_{l} \left( i \right) {\text{and}} n_{p} \left( i \right)$ are the number of voxels located with points and the number of all voxels, respectively; *n* is the number of vertical layers.

PAI was equal to the sum of PAD in the horizontal layers (Eq. 4).

4 $${\text{PAI}} = \mathop \sum \limits_{i = 0}^{i = n} PAD \times \Delta H$$

Similarly, 3DPI was calculated using Eq. 5:

5 $$3DPI = \mathop \sum \limits_{i = 0}^{i = n} \left( {\frac{{p_{i} }}{{p_{t} }}} \right)e^{{k\frac{{p_{cs} }}{{p_{t} }}}}$$

where *n* is the number of vertical layers; $p_{i}$ is the number of points located in layer *i*,$p_{t}$ is the total number of points in all layers; $p_{cs}$ is the sum of points above layer *i*; *k* is a given parameter, which is set as 1 according to Jimenez-Berni et al. [24].

Volume was calculated using Eq. 6:

6 $${\text{Volume}} = \Delta {\text{x}} \times \Delta {\text{y}} \times \Delta {\text{z}} \times \frac{{n_{l} }}{{n_{p} }}$$

where $\Delta {\text{x}}, \Delta {\text{y}}, {\text{and }}\Delta {\text{z}}$ are the length of bounding box of points in x, y, and z directions. $n_{l} {\text{ and }} n_{p}$ are the number of voxels located with points and the number of all voxels, respectively.

#### Phenotypic trait extraction at individual plant level

At the individual plant level (Figs. 1c, 2b), each plot was first segmented into individual maize plants using a faster RCNN-based deep learning method (FRCNN) [25]. The segmented results were visually checked and refined with the LiDAR 360 software to ensure the accuracy of the following phenotypic trait extraction. Finally, 138 individual plants were segmented and used to extract phenotypic traits, including maximum individual height (height), crown size, ratio of crown size to height (CHR), canopy cover, PLA, PAI, 3DPI, and volume. Crown size was defined as the maximum length of the crown in all directions; CHR was calculated by dividing crown size by height; the other phenotypic traits were calculated with the same formulas used at plot level.

#### Phenotypic trait extraction at leaf group level

At leaf group level (Figs. 1c, 2c), the above-segmented 138 individual plants were further segmented into 138 leaf groups and 138 stems using a voxel-based convolution neural network (VCNN) method [26]. The 138 separated leaf groups were used to extract phenotypic traits, including height, crown size, CHR, canopy coverage, PLA, PAI, 3DPI, and volume of leaves using the abovementioned methods.

#### Phenotypic trait extraction at organ level

At the stem level (Figs. 1c, 2c), the 138 stems were used to extract stem height, diameter, PAI, 3DPI, and volume. Stem height, PAI, 3DPI, and volume were calculated using the abovementioned methods. Stem diameter was calculated using a circle fitting method. The stem points were sliced into 3 equal horizontal layers. In each layer, points were projected into the X–Y plane and fitted with a circle using the least-squares method, which returned the diameter of the circle. The diameters extracted for the three layers were averaged to derive the stem diameter. Based on the stem diameter and stem height, stem volume was calculated using the cylindrical volume formula.

At the individual leaf level (Figs. 1b, 2d), the scanned plot was segmented into individual plants using FRCNN, which were then segmented into individual leaf samples using VCNN and refined with the LiDAR360 software [31]. Leaf length, leaf mean width, leaf area, projected leaf length (PLL), PLA, PAI, 3DPI, and volume were calculated for each individual leaf sample. Among them, leaf length, leaf mean width, leaf area were calculated using a skeleton-based method [31]. PLL and PAL were the leaf length and leaf area calculated with the projection points at the X–Y projection plane. The other phenotypic traits were calculated using the same methods as used at plot level. All methods were implemented using Python 3.6 software according to the definitions/formulas.

The extracted phenotypic traits were matched with field measurements for model building at plot, individual plant, leaf group, stem, and individual leaf levels. The matched sample size at plot, individual plant, leaf group, and stem are 55, 138, 138, and 138, respectively (Table 3). At the individual leaf level, it was a very time-consuming and difficult task to match all individual leaf measurements with lidar measurements, and scenarios of broken leaves or too small leaves may even make the matching process of certain leaves become an impossible task. In this study, we selected 59 individual leaf samples instead of all leaves, and matched them with lidar data for individual leaf level biomass estimation. These leaf samples covered a leaf length from 0.320 m to 1.022 m and a leaf width from 0.039 m to 0.200 m (Table 3), ensuring the representativeness of the selected samples.

### Methods for estimating biomass from terrestrial lidar data

To systematically answer which method and which phenotypic trait are best for biomass estimation, we used all phenotypic traits to build regression models, including simple regression (SR), stepwise multiple regression (SMR), artificial neural network (ANN), and random forest (RF). Two forms of SR were implemented. One was the simple linear regression (SLR) based on the raw prediction variables and an independent variable, the other was the log-transformed simple regression (LSR) based on log transformed dependent and log transformed independent variables.

#### SR method

The SR method was selected due to its simple procedure and strong fitting ability, which usually contains only one independent variable. In this study, both SLR and LSR were used to predict maize biomass at different levels. Models with the highest coefficient of determination were treated as the best models, and their corresponding independent variables were selected as the best traits. The SLR (Eq. 7) and LSR (Eq. 8) formulas are defined as:

7 $$B = \alpha \times V + \beta$$

8 $$ln\left( B \right) = \alpha \times Ln\left( V \right) + \beta$$

where *B* is the predicted biomass (g), *V* is the phenotypic trait extracted from terrestrial lidar data, $\alpha$ and $\beta$ are regression parameters.

#### SMR model

SMR is a multiple linear regression method that can automatically select strong correlated independent variables according to their influence on dependent variables. The influence of the variables can be evaluated with criteria such as the Akaike information criterion (AIC) and F-tests. The SMR has three different approaches, i.e., forward elimination, backward elimination, and bidirectional elimination. In this study, the backward elimination approach according to the AIC criterion was selected, which means that all variables were used in the first step and a variable was eliminated if it could not reduce the AIC value [32]. The SMR model was constructed and validated using the ‘stepwise’ package in R software.

#### ANN regression model

ANN, inspired by biological neural networks, is a multi-layer fully connected structure for nonlinear feature learning, which consists of an input layer, one or more hidden layers, and an output layer. In this study, the number of hidden layers was set to 1, because one hidden layer is sufficient for solving biomass regressions in most cases [33, 34]. In addition to the number of hidden layers, the number of neurons in each layer is also an important parameter. The number of neurons in the input layer and output layer are determined by the number of independent variables and dependent variable, respectively. However, the number of neurons in the hidden layer is uncertain though important. To find an optimal number of neurons in the hidden layer, a trial-and-error method [35] was used to find the best ANN model with hidden neuron numbers from 1 to 20 with an interval of 1. During the construction of the ANN model, all variables needed to be normalized. After we selected the best model, parameter importance was analyzed using the connection weight analysis method [36]. Both the model construction and importance analysis were implemented using the R ‘NeuralNet’ package.

#### RF regression model

RF is an ensemble learning method used for classification and regression. RF constructs multiple decision trees during the training phase and outputs the mode class and mean value of the trained trees for classification and regression, respectively. There are two important parameters, *ntree* and *mtry*, that influence the performance of a RF model [37]. The parameter *ntree* is referred as the number of trees to grow in the RF model, which should be set large when the sample size is large. The parameter *mtry* represents the number of variables selected for splitting at each node, which should be set smaller than the total number of independent variables [38]. Due to their uncertain influence, we used a grid searching method to find an optimal combination of *ntree* and *mtry* to maximize the model accuracy. In this study, *ntree* was varied from 100 to 1000 with an interval of 100, *mtry* was set from 1 to the maximum number of variables with an interval of 1. Each *ntree* was combined with each *mtry* to run the RF models. The model that had the best prediction accuracy was selected to determine the best *ntree* and *mtry*. In addition, parameter importance was analyzed using the percentage increase in the mean-squared error (%IncMSE). A larger  %IncMSE denotes that a variable is more important [39]. Both the model construction and importance analysis were implemented using the R ‘randomForest’ package.

### Accuracy assessment

To evaluate the performance of different models, data were split into 70% training/fitting data and 30% testing data. The training data were used to train/fit the abovementioned models. Testing data were used to evaluate model accuracy by comparing the model predicted and field-measured biomass at different levels based on the determination coefficient (R^2^) and root-mean-squared error (RMSE).

9 $$R^{2} = 1 - \frac{{\mathop \sum \nolimits_{i = 1}^{n} \left( {B_{i} - \widehat{{B_{i} }}} \right)^{2} }}{{\mathop \sum \nolimits_{i = 1}^{n} \left( {B_{i} - \overline{{B_{i} }} } \right)^{2} }}$$

10 $${\text{RMSE}} = \sqrt { \frac{1}{n}\mathop \sum \limits_{i = 1}^{n} \left( {B_{i} - \widehat{{B_{i} }}} \right)^{2} }$$

where n is the number of testing samples; $B_{i}$ represents the field-measured biomass; $\widehat{{B_{i} }}$ represents the predicted biomass by a regression model; $\overline{{B_{i} }}$ is the average value of all $B_{i}$.

To analyze the best model and phenotypic traits at different levels, we built SR models using all available phenotypic traits at each level. However, only the best height quantile trait that had the highest correlation with the field biomass according to the SR models was kept for building the multivariable models (i.e., SMR, RF, ANN) with other traits. According to the highest prediction accuracy (R^2^), the best models were selected. Moreover, variables importance was analyzed for different models. The most important variables were selected according to the best SR model. Common important variables in the multivariable models (SMR, ANN, and RF) were also selected.

## Results

### Biomass estimation at plot level

**Fig. 3:**
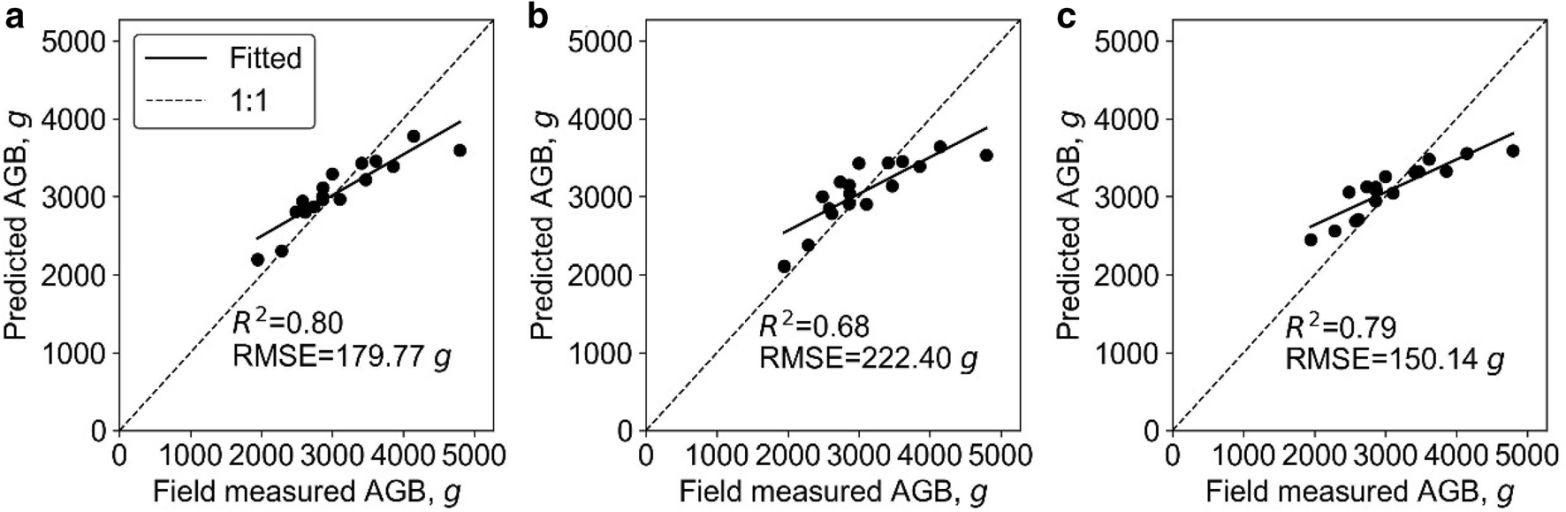
Correlations between predicted and field-measured biomass at plot level. **a**–**c** show the results derived from the SMR model, the ANN model, and the RF model, respectively. Note that the solid line represents the fitted line and the dashed line represents the 1:1 line

**Fig. 4:**
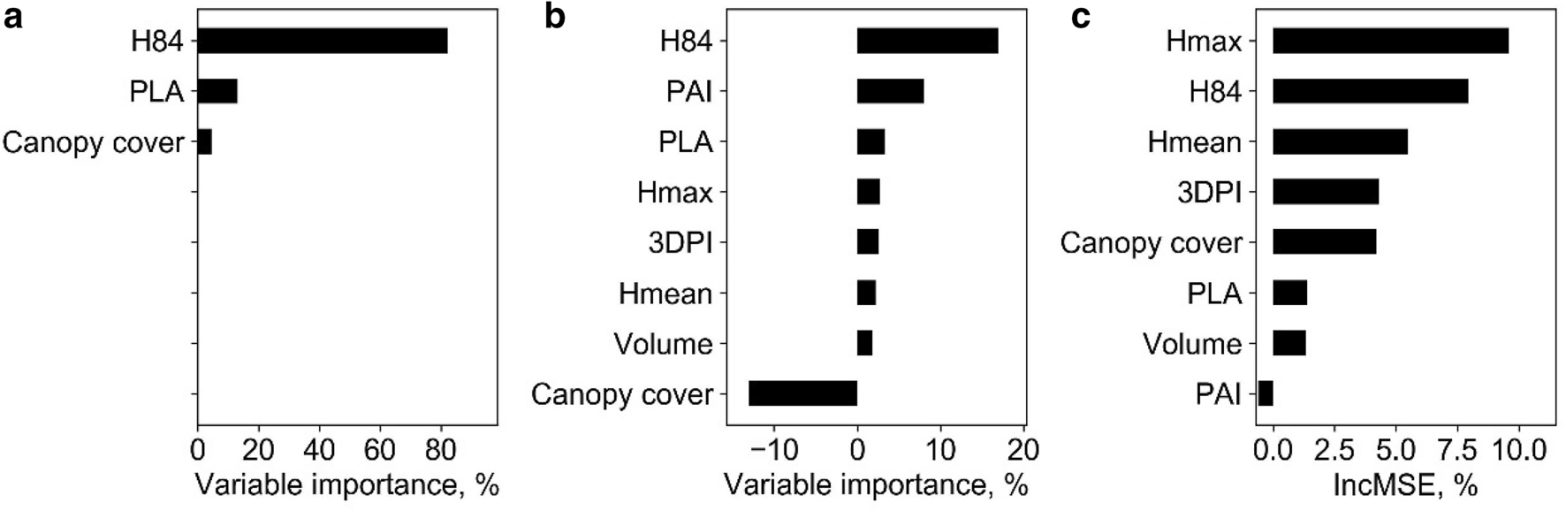
Variable importance analysis for **a** the SMR model, **b** the ANN model, and **c** the RF model at plot level. Note that H84, H_max_, H_mean_, PLA, PAI, and 3DPI represent the 80% quantile height, max point height, mean point height, projected leaf area, plant area index, and three-dimensional profile index, respectively

At plot level, the SR models, including SLR and LSR, were fitted to predict plot biomass using each extracted phenotypic trait. All SR models built with height-related variables were highly significant (p < 0.01) and the model built with 3DPI was significant (p < 0.05), while other variables resulted in non-statistically significant models (Table 4). In most cases, the LSR model slightly outperformed SLR. The best LSR model and SLR model were both built with variable H84, which had R^2^ of 0.79 and 0.80, respectively. SMR, ANN, and RF models were fitted with the phenotypic trait that showed the best R^2^ in SR models (i.e., H84) and other phenotypic traits. The hidden size of ANN was 17, and the *ntree* and *mtry* of RF were 200 and 2, respectively. The R^2^ (RMSE) of SMR, ANN, and RF were similar to the ones obtained by the SR models, and were 0.80 (179.77 g), 0.68 (222.40 g), and 0.79 (150.14 g), respectively (Fig. 3). Moreover, we found that these models all overestimated and underestimated at low and high biomass levels, respectively. In addition, the variable importance analysis of the SMR model showed that the important variables were H84, PLA, and canopy cover (Fig. 4). The most important variables in the ANN model were H84, canopy cover, PAI, and PLA. As for RF, the most important variables were mainly height related, including H_max_, H84, and H_mean_.

**Table 4:** Coefficients of determination (R^2^) and root mean square errors (RMSE) between the predicted and field-measured biomass at plot level using simple regression methods

Variable	Simple linear regression		Log transformed simple regression	
	R^2^	RMSE, g	R^2^	RMSE, g
H_max_	0.45**	540.88	0.45**	534.22
H_mean_	0.59**	478.53	0.59**	461.92
H99	0.59**	487.22	0.60**	478.55
H98	0.65**	463.21	0.66**	452.51
H97	0.67**	450.19	0.68**	438.69
H96	0.69**	435.83	0.70**	423.29
H95	0.70**	424.75	0.71**	411.57
H94	0.71**	418.13	0.72**	404.22
H93	0.72**	409.72	0.74**	394.65
H92	0.74**	400.38	0.75**	383.87
H91	0.75**	392.48	0.76**	374.88
H90	0.76**	387.18	0.77**	368.72
H89	0.76**	384.09	0.78**	364.9
H88	0.77**	383.10	0.78**	363.52
H87	0.77**	381.70	0.78**	361.71
H86	0.78**	377.77	0.79**	357.07
H85	0.78**	374.82	0.80**	353.16
*H84*	0.79**	*374.59*	0.80**	*352.12*
H83	0.79**	376.32	0.8**	353.34
H82	0.78**	379.15	0.8**	356.11
H81	0.78**	382.18	0.79**	359.13
H80	0.78**	384.22	0.79**	361.04
Canopy cover	0.01	697.11	0.01	706.48
PLA	0	718.57	0	733.73
Volume	0.01	725.22	0.02	730.90
PAI	0.01	697.10	0	718.56
3DPI	0.24*	615.58	0.24*	626.41

Italic values indicate the most important variable and the corresponding prediction accuracy (i.e., R2 and RMSE) of simple regression models (i.e., SLR and LSR)

* p < 0.05; ** p < 0.01

Overall, the SMR model had the highest biomass prediction accuracy at plot level, followed by LSR, RF, SLR, and ANN. However, SR models had the highest RMSE (Fig. 3). The most important predictors were height related variables (i.e., H84, H_max_, H_mean_), followed by canopy cover, PLA, and PAI.

### Biomass estimation at individual plant level

**Fig. 5:**
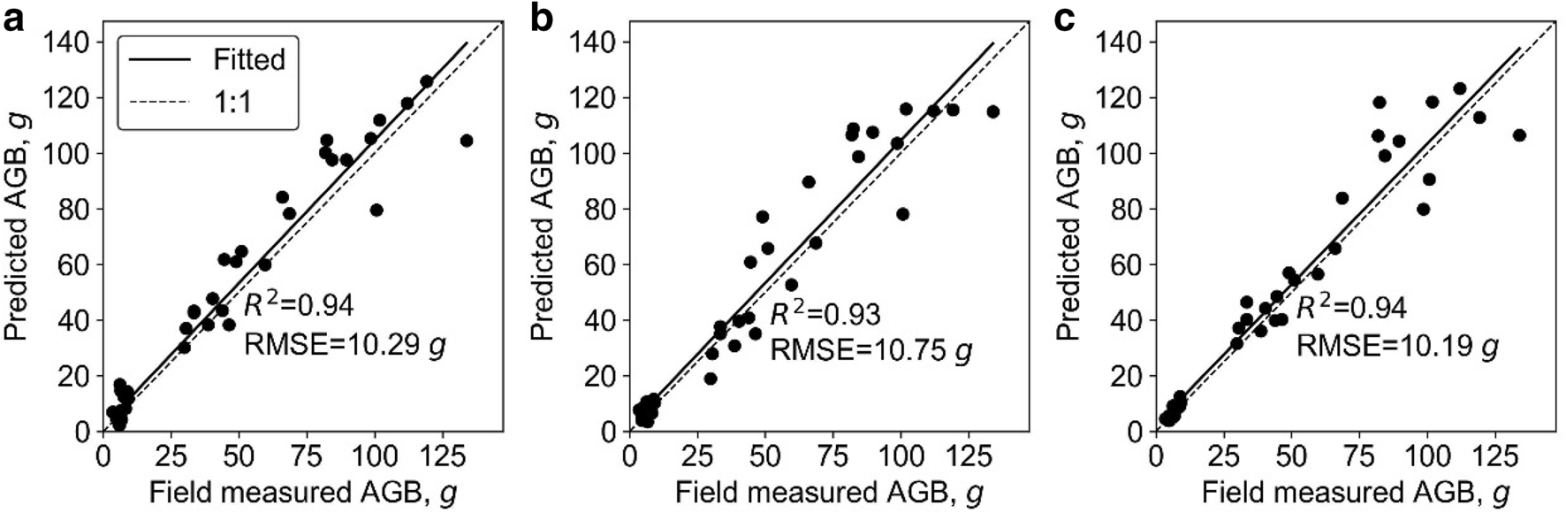
Correlations between predicted and field-measured biomass at individual plant level. **a**–**c** show the results derived from the SMR model, the ANN model, and the RF model, respectively. Note that the solid line represents the fitted line and the dashed line represents the 1:1 line

**Fig. 6:**
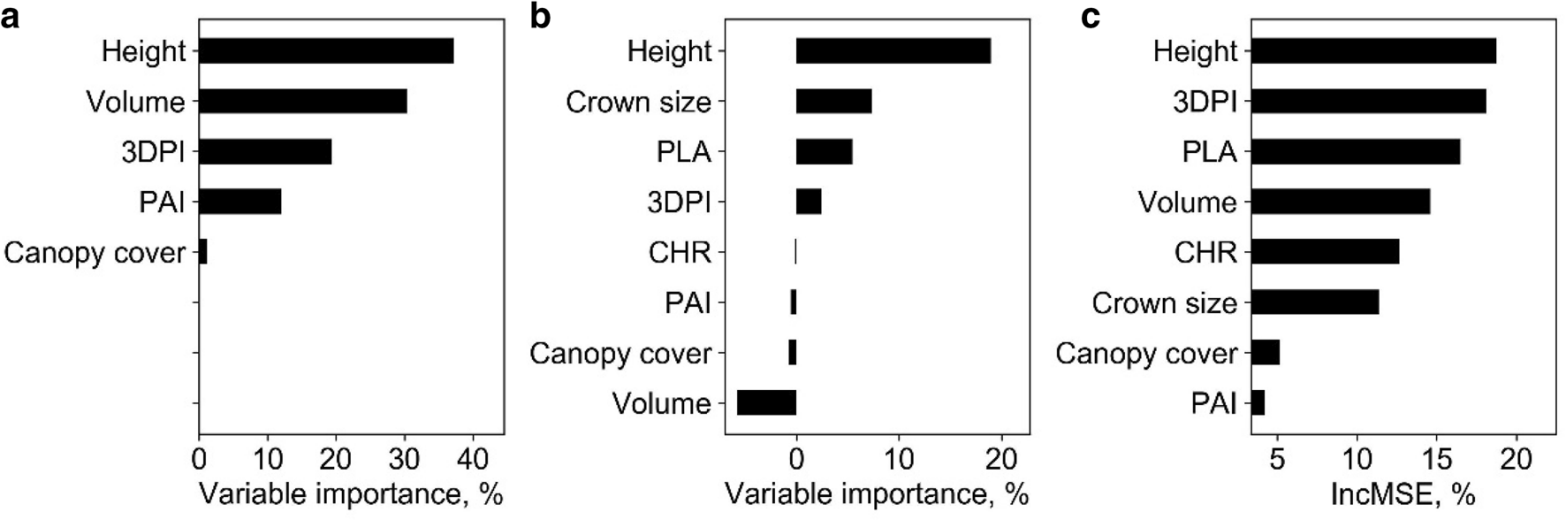
Variable importance analysis for **a** the SMR model, **b** the ANN model, and **c** the RF model at individual plant level. Note that PAI, PLA, 3DPI, and CHR represent the plant area index, projected leaf area, three-dimensional profile index, and ratio of crown size to height, respectively

At individual plant level, all SR models were highly significant (p < 0.01) except for the models built with canopy cover (Table 5). The best LSR model and SLR model were both built with the height variable and had R^2^ of 0.93 and 0.96, respectively. Meanwhile, the SMR, ANN, and RF models were fitted with all the extracted phenotypic traits. The hidden size of ANN was 2, and the *ntree* and *mtry* of RF were 600 and 2, respectively. The R^2^ (RMSE) of SMR, ANN, and RF were also high, and were 0.94 (10.29 g), 0.93 (10.75 g), and 0.94 (10.19 g), respectively (Fig. 5). We found that these models all fitted close to the 1:1 line without obvious saturation effects. In addition, the variable importance analysis of the SMR model showed that the most important variables were height, volume, and 3DPI (Fig. 6). The most important variables in the ANN model were height, crown size, and volume. As for the RF, the most important variables were height, 3DPI, and PLA.

**Table 5:** Correlations between predicted and field-measured biomass at individual plant level using simple regression methods

Variable	Simple linear regression		Log transformed simple regression	
	R^2^	RMSE, g	R^2^	RMSE, g
*Height*	0.93**	*10.78*	0.96**	*9.13*
Crown size	0.64**	23.24	0.72**	21.98
CHR	0.66**	22.87	0.65**	23.69
Canopy cover	0.06	37.47	0.06	41.27
PLA	0.71**	21.91	0.67**	22.42
Volume	0.83**	17.24	0.81**	18.12
PAI	0.43**	31.39	0.33**	37.72
3DPI	0.64**	23.24	0.78**	19.6

Italic values indicate the most important variable and the corresponding prediction accuracy (i.e., R2 and RMSE) of simple regression models (i.e., SLR and LSR)

* p < 0.05; ** p < 0.01

Overall, the LSR model had the highest biomass prediction accuracy at individual plant level, followed by RF, SMR, ANN, and SLR, which also had high accuracy (Fig. 5). The most important predictors were height, 3DPI, and volume.

### Biomass estimation at leaf group level

**Fig. 7:**
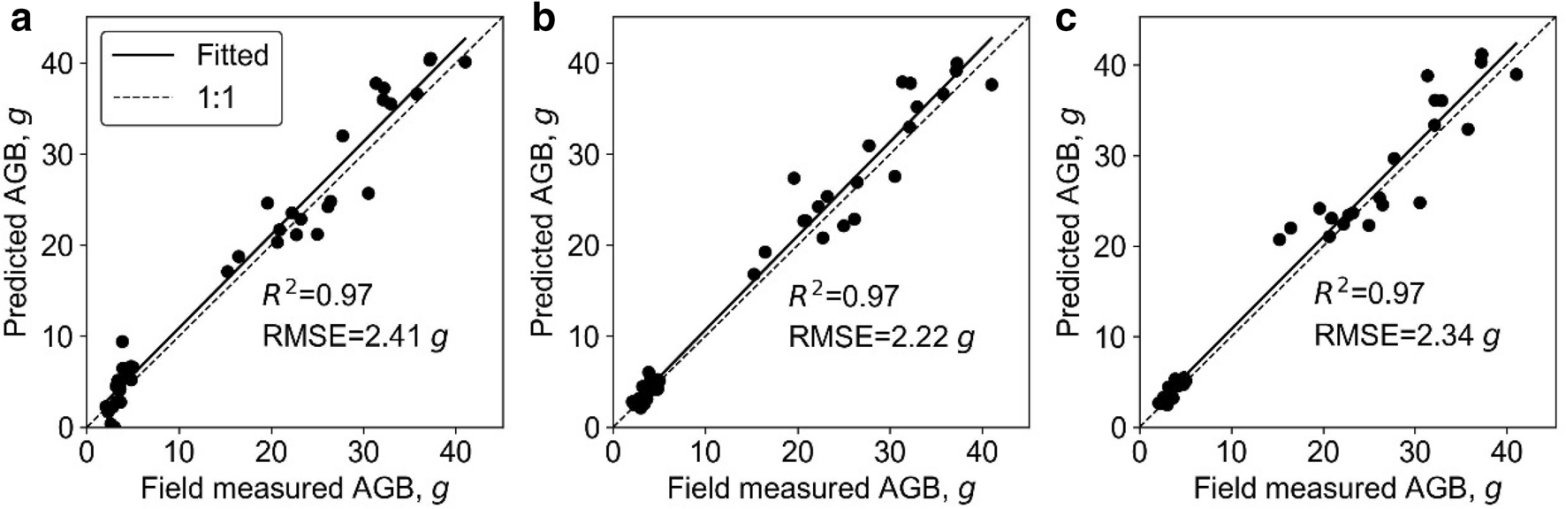
Correlations between predicted and field-measured biomass at leaf group level. **a**–**c** represents the results derived from the SMR model, the ANN model, and the RF model, respectively. Note that solid line represents the fitted line and the dashed line represents the 1:1 line

**Fig. 8:**
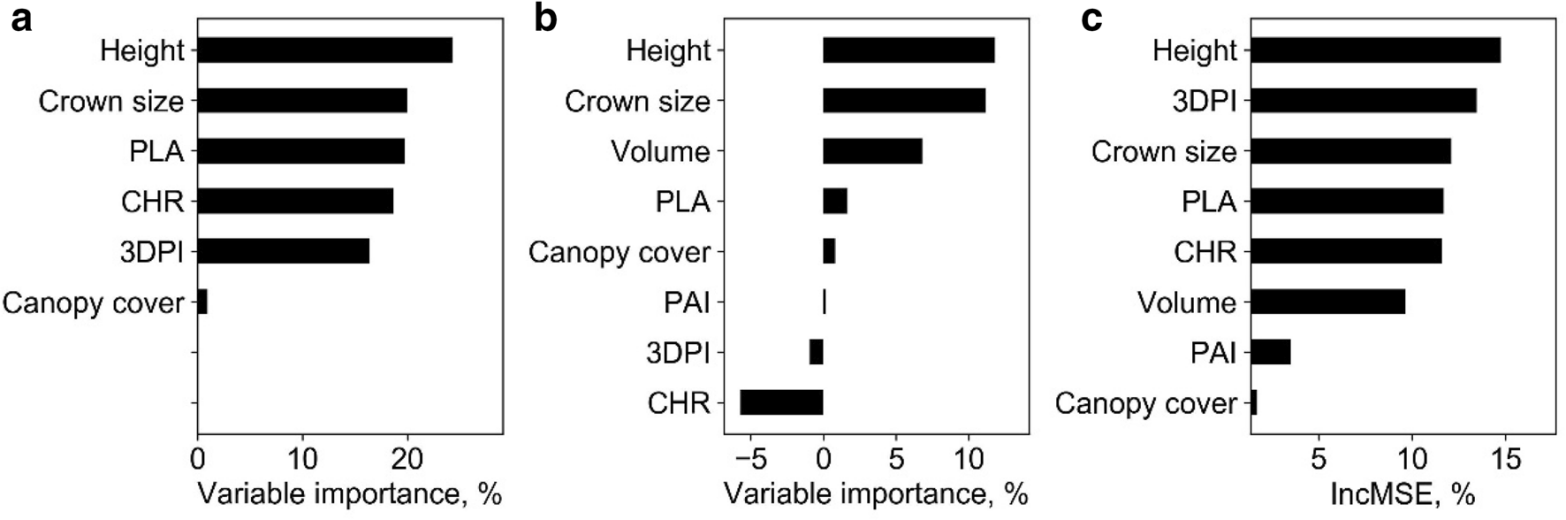
Variable importance analysis for **a** the SMR model, **b** the ANN model, and **c** the RF model at leaf group level. Note that PLA, CHR, 3DPI, and PAI represent the projected leaf area, ratio of crown size to height, three-dimensional profile index, and plant area index, respectively

At leaf group level, all SR models were highly significant (p < 0.01) except models built with canopy cover (Table 6). The best LSR model and SLR model were built with height and 3DPI variables and had R^2^ of 0.95 and 0.92, respectively. Meanwhile, SMR, ANN, and RF models were fitted with all phenotypic traits. The hidden size of ANN was 1, and the *ntree* and *mtry* of RF were 400 and 2, respectively. The R^2^ (RMSE) of SMR, ANN, and RF were also high, and were 0.97 (2.41 g), 0.97 (2.22 g), and 0.97 (2.34 g), respectively (Fig. 7). These models all fitted closely to 1:1 line. In addition, the variable importance analysis of the SMR model showed that the important variables were height, crown size, and PLA, etc (Fig. 8). The most important variables in the ANN model were height, crown size, and CHR. As for RF, the most important variables were height, 3DPI, and crown size.

**Table 6:** Correlations between predicted and field-measured biomass at leaf group level using simple regression methods

Variable	Simple linear regression		Log transformed simple regression	
	R^2^	RMSE, g	R^2^	RMSE, g
*Height*	0.95**	*3.32*	0.89**	5.37
Crown size	0.69**	7.76	0.69**	7.18
CHR	0.65**	8	0.54**	10.86
Canopy cover	0.02	13.37	0.02	13.16
PLA	0.87**	4.86	0.84**	5.28
Volume	0.84**	5.34	0.81**	6.1
PAI	0.48**	9.98	0.49**	9.49
3DPI	0.8**	6.09	0.92**	*3.77*

Italic values indicate the most important variable and the corresponding prediction accuracy (i.e., R2 and RMSE) of simple regression models (i.e., SLR and LSR)

* p < 0.05; ** p < 0.01

Overall, the SR, SMR, ANN, and RF models had similar high accuracy (R^2^ ≥ 0.95) at leaf group level (Fig. 7). The most important predictors were height, crown size, and 3DPI.

### Biomass estimation at organ level

**Fig. 9:**
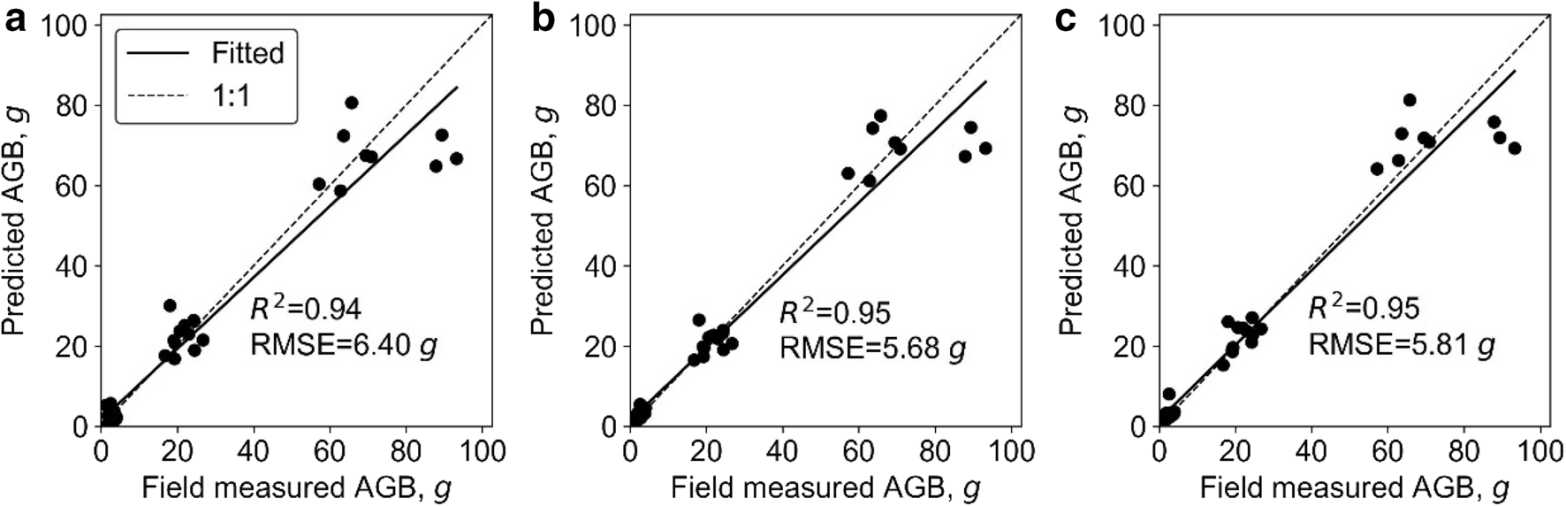
Correlations between predicted and field-measured biomass at stem level. **a**–**c** represent the results derived from the SMR model, the ANN model, and the RF model, respectively. Note that the solid line represents the fitted line and the dashed line represents the 1:1 line

**Fig. 10:**
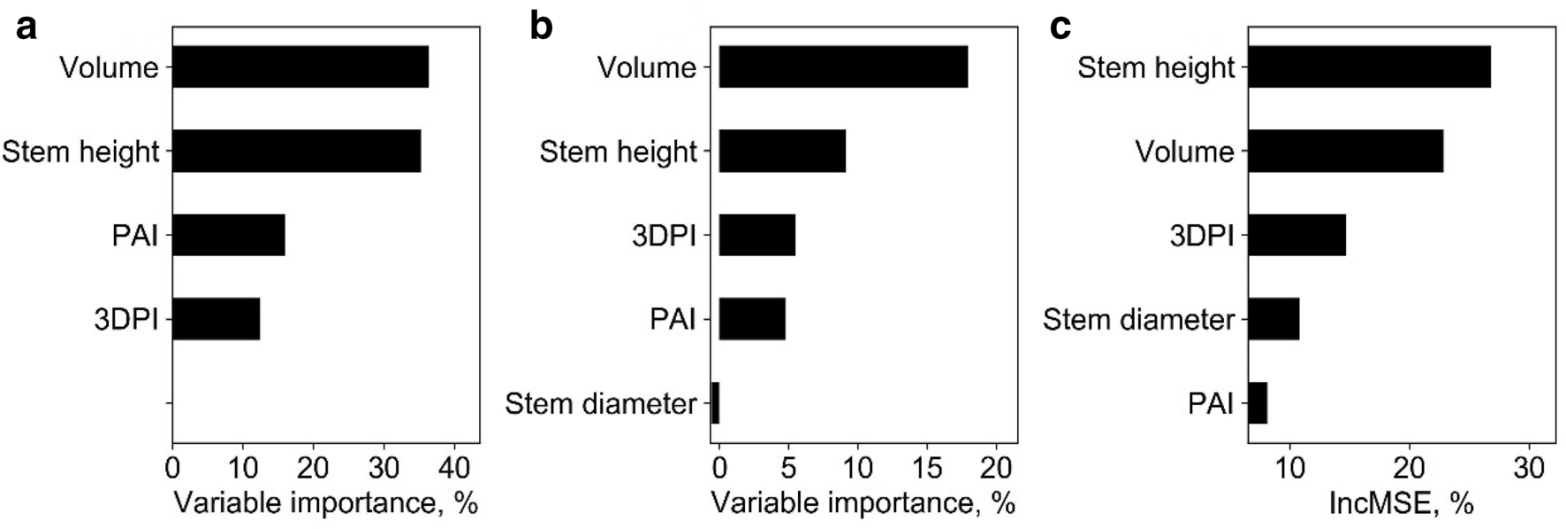
Variable importance analysis for **a** the SMR model, **b** the ANN model, and **c** the RF model at stem level. Note that PAI and 3DPI represent the plant area index and three-dimensional profile index, respectively

At stem level, SR models, all SR models were highly significant (p < 0.01) (Table 7). The best LSR model and SLR model were both built with stem height variable and had R^2^ of 0.93 and 0.94, respectively. Meanwhile, the SMR, ANN, and RF models were fitted with all phenotypic traits. The hidden size of ANN was 2, and the *ntree* and *mtry* of RF were 800 and 2, respectively. The R^2^ (RMSE) of SMR, ANN, and RF were also high, and were 0.94 (6.40 g), 0.95 (5.68 g), and 0.95 (5.81 g), respectively (Fig. 9). These models all underestimated at high biomass, but they did not overestimate at low biomass. In addition, the variable importance analysis of the SMR model showed that the important variables were volume, stem height, and PAI (Fig. 10). The most important variables in the ANN model were volume, stem height, and 3DPI. As for RF, the most important variables were stem height, volume, and 3DPI.

**Table 7:** Correlations between predicted and field-measured biomass at stem level using simple regression methods

Variable	Simple linear regression		Log transformed simple regression	
	R^2^	RMSE, g	R^2^	RMSE, g
Stem diameter	0.76**	13.99	0.80**	12.93
*Stem height*	0.93**	*8.14*	0.94**	*7.46*
Volume	0.90**	8.89	0.91**	8.63
PAI	0.51**	19.62	0.45**	21.38
3DPI	0.48**	20.19	0.69**	19.64

Italic values indicate the most important variable and the corresponding prediction accuracy (i.e., R2 and RMSE) of simple regression models (i.e., SLR and LSR)

* p < 0.05; ** p < 0.01

Overall, all models had high accuracy at stem level, of which the multivariable methods (i.e., SMR, ANN, and RF) were slight better (Fig. 9). The most important predictors were volume, stem height, 3DPI, and PAI.

**Fig. 11:**
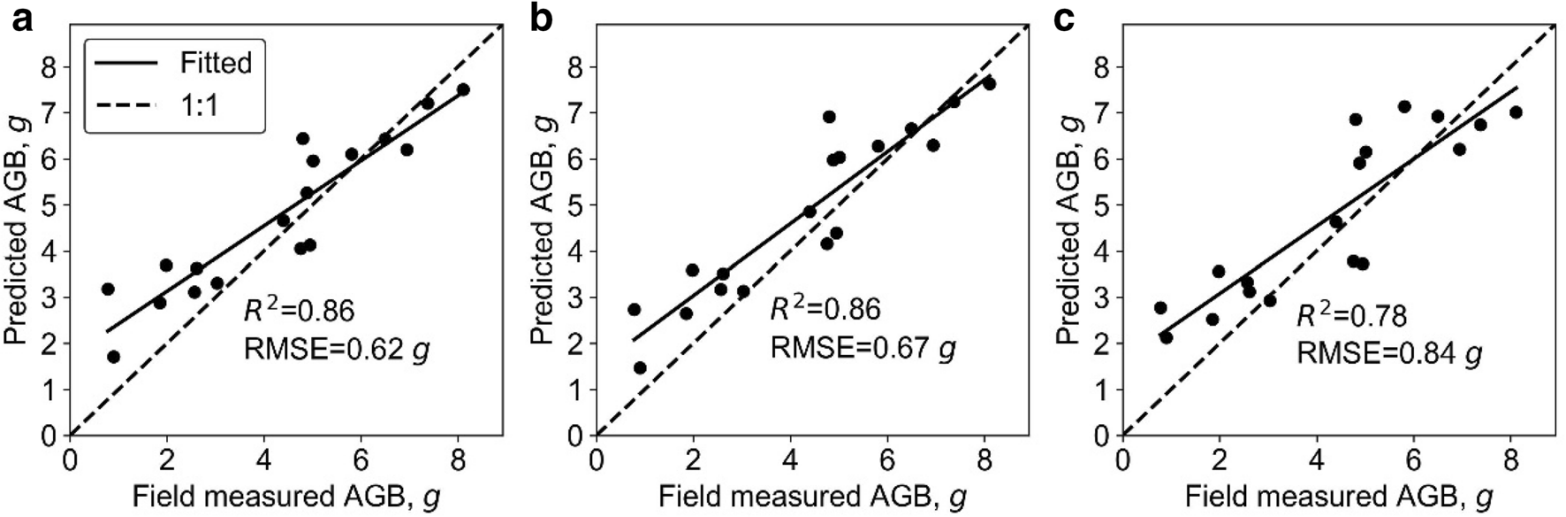
Correlations between the predicted and field-measured biomass at the individual leaf level. **a**–**c** represent the results derived from the SMR model, the ANN model, and the RF model, respectively. Note that solid line represents the fitted line and the dashed line represents the 1:1 line

**Fig. 12:**
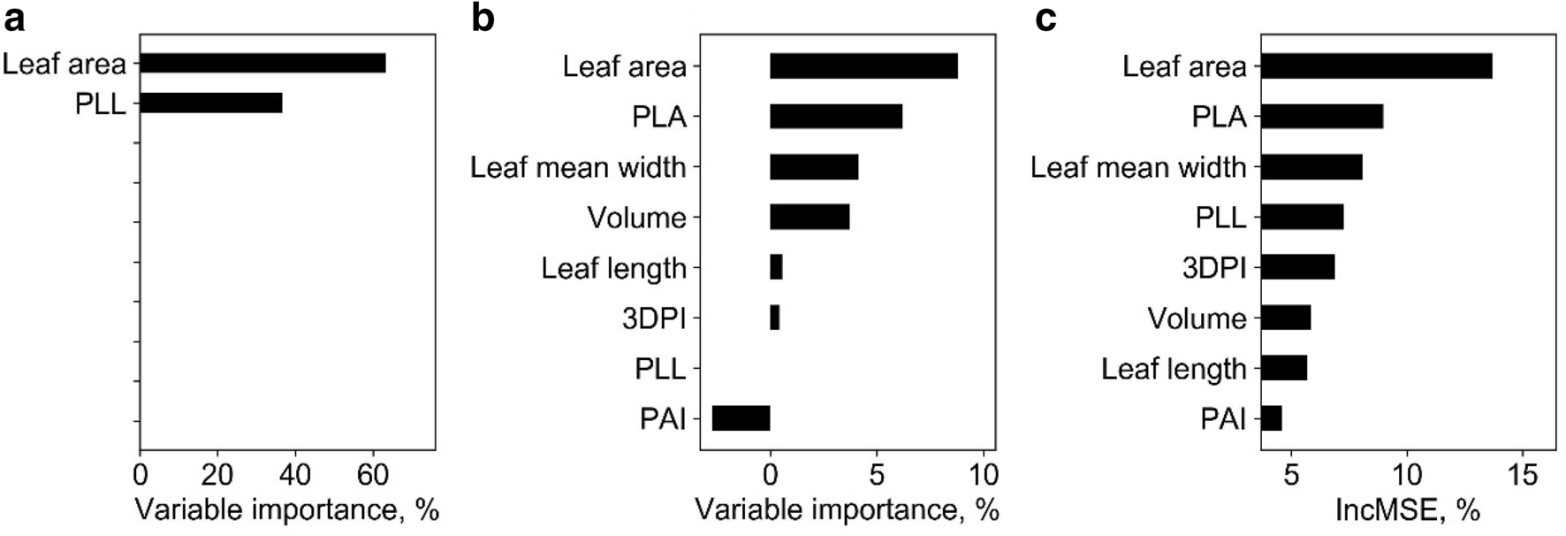
Variable importance analysis for **a** the SMR model, **b** the ANN model, and **c** the RF model at individual leaf level. Note that PLL, PLA, 3DPI, and PAI represent the projected leaf length, projected leaf area, three-dimensional profile index, and projected area index, respectively

At the individual leaf level, all SR models were highly significant (p < 0.01) except models built with PAI (Table 8). The best LSR model and SLR model were both built with the leaf area variable with R^2^ of 0.84 and 0.84, respectively. Meanwhile, SMR, ANN, and RF models were fitted with all the phenotypic traits. The hidden size of ANN was 20, and the *ntree* and *mtry* of RF were 400 and 2, respectively. The R^2^ (RMSE) of SMR, ANN, and RF were 0.86 (0.62 g), 0.86 (0.67 g), and 0.78 (0.84 g), respectively (Fig. 11). These models all overestimated and underestimated at low and high biomass, respectively. In addition, the variable importance analysis of the SMR model showed that the important variables were leaf area and PLL (Fig. 12). The most important variables in the ANN model were leaf area, PLA, and leaf mean width. As for RF, the most important variables were leaf area, PLA, and leaf mean width.

**Table 8:** Correlations between predicted and field-measured biomass at the individual leaf level using simple regression methods

Variable	Simple linear regression		Log transformed simple regression	
	R^2^	RMSE, g	R^2^	RMSE, g
Leaf length	0.76**	1.25	0.77**	1.13
Leaf mean width	0.67**	1.35	0.67**	1.3
*Leaf area*	0.84**	*1.02*	0.84**	*0.99*
Projected leaf length	0.49**	1.83	0.43**	1.76
PLA	0.79**	1.3	0.72**	1.37
Volume	0.61**	1.47	0.65**	1.38
PAI	0.12	2.11	0.04	2.1
3DPI	0.38**	1.87	0.39**	1.91

Italic values indicate the most important variable and the corresponding prediction accuracy (i.e., R2 and RMSE) of simple regression models (i.e., SLR and LSR)

* p < 0.05; ** p < 0.01

Overall, all models showed relatively high accuracies. The SMR model had the highest biomass prediction accuracy at the individual leaf level, followed by ANN, SR, and RF (Fig. 11). The most important predictors were leaf area, PLL, PLA, and leaf mean width.

## Discussion

### Importance of high-throughput phenotyping for biomass estimation

In this study, maize biomass estimation was based on accurate data and high-throughput phenotypic trait extraction algorithms. In recent years, lidar has proved the ability to provide data at millimeter-scale accuracy for high-throughput phenotyping [13, 14]. Meanwhile, an increasing number of studies have shown the potential of high-throughput phenotypic trait extraction for biomass estimation. For 1D phenotypic trait extraction, Jin et al. [31] found that the R^2^ between lidar and field-measured individual maize height, leaf length, leaf width, stem height, stem diameter and crown size were 0.91, 0.88, 0.81, 0.97, 0.65, and 0.96, respectively. Although for stem diameter the R^2^ was relatively low, the RMSE was only 0.01 m. For two dimensional (2D) and 3D phenotypic traits, Jin et al. [31] demonstrated the high correlation (R^2^ = 0.88) between volume and total leaf area. Other studies have shown that lidar can provide highly accurate estimates of canopy cover [24] and PAI [28]. Lidar data and algorithm development have enabled high-throughput phenotyping biomass estimation [24].

The development of deep learning further contributed to the implementation of high-throughput phenotyping imaging at fine scales, creating the opportunity to advance biomass estimation from a traditional plot level to finer levels. Deep learning methods have removed the key bottlenecks for high-throughput phenotypic traits extraction, which are individual plant segmentation and stem-leaf segmentation. Their accuracy and robustness have been discussed in our previous studies [25, 26, 31]. The F-score of individual segmentation reached 0.90 [25]. The overall classification accuracy of stem-leaf classification at instance level and point level were 1.0 and 0.96, respectively. The F-score of stem-leaf segmentation at instance level and point level were 0.91 and 0.89, respectively [26].

To summarize, advancements in high-throughput data and algorithms made it possible to estimate biomass at fine levels. In this study, high accurate lidar data were collected and processed based on deep learning methods. Moreover, we have manually refined the individual segmentation and stem-leaf segmentation results in the LiDAR360 software, which further improved the accuracy of the parameters in this study. Optimal parameter setting for calculating phenotypic traits (e.g., voxel size) were referred from related studies [24, 28], while the best parameters for the estimation models (i.e., ANN and RF) were determined through trial-and-error [35]. The highly accurate lidar data, state-of-the-art algorithms, interactive refinement, and best parameter setting lay a good foundation for constructing biomass estimation models.

### Best models and phenotypic traits for biomass estimation at different levels

At plot level, the best model for biomass estimation were SMR with H84, PLA, and canopy cover variables, followed by SR, RF, and ANN. The results were consistent with previous studies showing that multiple variable based methods are better than SR methods, and ANN may perform weakly with insufficient sample data [33, 34]. However, all the models had R^2^ of no more than 0.80, and they all showed a saturation effect. A possible reason is that the plot data were all collected during the mature stage, and had a small biomass range. Li et al. [6] also found that biomass estimation accuracy is high when using long-term data but is relatively low at any single growing stage. In addition, the important variables are consistent with previous findings in biomass estimation in grassland, agriculture, and forest fields [40–43]. The reason why 2D variables (i.e., canopy cover and PLA) were not significantly related with biomass is the symmetrical distribution of maize leaves. In the mature stage, the upper leaves of maize block the lower leaves, causing only a small difference in canopy coverage and PLA under different biomass levels. The importance of 3D variables may be influenced by the points quality. In this study, the mean plot height was 2.46 m, which prevented the laser from fully penetrating the canopy and resulted in the spare point density at the bottom of the plants (Additional file 1: Figure S1). The sparse point density caused the underestimation and uncertainty of the 3D related traits, including PAI, 3DPI, and volume. However, in our study we still found 3DPI to be significantly related to biomass, which means that 3D related traits should have the potential to estimate biomass because they contain height information. By contrast, height is a 1D phenotypic trait, which can be accurately extracted. Therefore, height related variables are always highly significantly related to biomass. However, the best variable in this study was H84 rather than a variable like H_max_ or H_mean_. The possible reason is that the height of individual maize plant strongly differs in each plot, causing H_max_ cannot represent the height property at plot level as suggested by previous studies [24]. H_mean_ is the mean point height value rather than the mean individual height, and thus also not the best estimation variable. In summary, 3D variables are not robust biomass estimators when data quality is poor, 2D variables tend to saturate early, while 1D height-related variables are the most reliable. The reason why the multivariable methods did not outperform the SR methods may be that the 2D and 3D variables are not robust at the mature stage.

At individual plant level, the best model for biomass estimation was the LSR with the height variable. Besides, all models performed well with R^2^ higher than 0.93 and have no saturation effect. The no saturation effect and the highly significant correlations between biomass with all variables except canopy cover may benefit from multi-temporal data and accurate parameter extraction from highly accurate terrestrial lidar data (Fig. 1a). The reason why canopy cover was not significantly related with biomass at the individual plant level is that canopy cover of an individual plant is normalized by its bounding box area, and is almost a constant even though the PLA increases with biomass. Overall, due to the high accuracy of the data used in this study, most 3D (e.g., volume, PAI, and 3DPI) and 2D variables were significant. However, the 1D height variable was still the most important at the individual level.

At leaf group level, the best models for biomass estimation were multi-variable models (R^2^ = 0.97). Besides, all models had high performances with R^2^ higher than 0.93 and had no saturation effect. The SR results showed that all variables were closely related to biomass except canopy cover, which was similar to findings at individual plant level. This might be reasonable because height has strong correlations with both leaf size and number of leaves, which are the two direct factors influencing the biomass at leaf group level. However, the most important variable in the LSR model was 3DPI, followed by height, suggesting that 3D variables may play as important role as 1D height at the leaf group level.

At the stem level, all models had a high performance with R^2^ greater than 0.93. The SR results show that all variables were highly significantly related with biomass (Table 6). However, the multi-variable models showed a slight underestimation. A possible reason is that the stem may be under-segmented in the upper part due to the aggregation of the top leaves in the late growth stages. From the variable importance analysis, volume was as important as stem height, which is consistent with previous findings that lidar-derived stem volume is strongly correlated to biomass [44].

At the individual leaf level, the best model for biomass estimation was SMR. However, the R^2^ of all models were no greater than 0.9. This may be because the parameter extraction accuracy at individual leaf level was not as accurate as at the stem and leaf group levels. Meanwhile, the saturation effect may be caused by variations in leaf water content. Usually, leaf water content decreases with the increase of leaf age. In this study, the ratio of wet weight to dry weight was estimated using leaves in the maturing stage, which may lead to overestimations to the biomass of young leaves and underestimations to the biomass of matured leaves. From the variable importance analysis, in the SR model all variables were highly significantly related with biomass, except PAI. Because PAI was proposed for an individual plant or plot, it may be not an appropriate variable at the individual leaf level. In contrast, leaf area, as a combination variable of leaf length and leaf width, is the most important variable.

From a comprehensive view at all different levels, we found that (1) R^2^ at the leaf group level were higher than that at individual plant level and group level, which indicates that terrestrial lidar data can be used for biomass estimation at fine levels. The pattern is similar to that of large level biomass estimation, in which the relative error of biomass estimation decreased with the decrease of plot size [45]. As state-of-the-art parameter extraction algorithms mature and become more widely used, the aggregation of fine-level biomass measurements is expected to yield more accurate biomass than at the plot level. In this study, we used the lidar measurements in 2018 to estimate the plot-level biomass by first estimating the biomass at the individual plant level, and then summing up individual measurements at each plot to generate plot-level biomass estimations. Results showed that the mean absolute error by using this method is 8.56 g/m^2^, comparing to a value of 28.94 g/m^2^ using the regression-based method. However, in practice, using regression-based method for plot-level biomass estimation is still the most commonly used because it has a higher efficiency and satisfying accuracy. Using individual plant-based method requires to segment individual plants first, which is a very time-consuming task in large-scale applications and may even be difficult to be used in fields with high plant density. Although biomass estimation accuracy seems to be higher at fine levels, the biomass estimation accuracy at the individual leaf level is not yet the highest, which may rely on the further improvements in individual leaf segmentation accuracy and parameter extraction accuracy. (2) In this study, all models had a similar high performance. However, the SR method is recommended because it requires only one independent variable. That may explain why traditional allometric functions are widely applied for forest biomass estimation [19, 20]. (3) As for parameter importance, height-related variables were the most important variables at plot, individual plant, leaf group, and stem levels. By contrast, previous studies found that optical vegetation indexes for crop biomass estimation changed with the growth stage and sampling data [8]. Therefore, lidar may provide more robust variables for fine level biomass estimation because lidar can acquire high accurate height information. Moreover, because height is an easily available variable at plot or larger level, which makes biomass estimation from lidar at large scale also feasible [6]. At the individual leaf level, individual leaf height was not considered since there is no meaningful definition for such parameter, but we found that leaf area was the most important. In addition, 3D variables, such as volume and 3DPI, are important for biomass estimation at stem, leaf, and individual plant levels. These height-related variables and 3D variables are hard to acquire through traditional field measurement or 2D image-based methods, suggesting that lidar may be particularly advantageous at fine levels.

### Contributions and future work

This study demonstrated the high accuracy of terrestrial lidar data and various regression models for biomass estimation from plot to individual leaf levels. Meanwhile, we explored the best models and phenotypes for biomass estimation at different levels through the comparison of different model performances for maize biomass estimation and parameter importance analysis. However, there are still some issues and researches that need to be illustrated and explored in the future. First, there may be autocorrelations between various predictors used in this study. One of the major goals of this study is to explore the variable importance for biomass estimation. The recognized physical meaningful variables with strong correlations with maize biomass can be used to guide users to measure phenotypes for biomass estimation in the future. Usually, methods such as principal component analysis for eliminating variable autocorrelations may remove the physical meanings of parameters, and make it hard to interpret. This is one of the major reasons that we decided to use the direct lidar measured variables with physical meaning to perform the analysis. This is also a commonly seen approach in similar studies that aim to evaluate the performance of different structural traits on functional trait estimations [33]. Second, although the biomass at plot level had a certain variability, it was all collected at the mature stage. In the future, time-series of biomass data at plot level need to be collected to better resolve the saturation effect. Third, recent studies have also demonstrated the feasibility of estimating canopy leaf water content using the backscatter coefficient derived from full-waveform terrestrial laser scanning data [46, 47]. In the current study, we did not have full-waveform terrestrial lidar data. In future, we will further investigate whether we can find a calibration function to remove the effect of water content from biomass estimation with 3D lidar data. Moreover, a comparison of the accuracy of biomass estimation using hyperspectral and lidar data may also help to illustrate the saturation effect at the individual leaf level and give an insight on biomass estimation with both structure and spectral information [5]. Fourth, matching individual leaves from the field and lidar data is a very time-consuming and challenging task because leaves might be broken in the packaging processes and too small to be found in lidar data. In this study, we randomly sampled 59 leaf samples and matched with field data. Although the leaf samples are representative, we will further collect more small leaf samples by acquiring high quality lidar data with better scanning settings. Finally, besides to scanning setting, more factors (e.g., sensor mode and point density) that influence the accuracy of biomass estimation are worth more investigations in future studies.

## Conclusions

Previously, terrestrial lidar data has been rarely used for crop biomass estimation, especially at individual plant or even finer levels because of the limited data and algorithms. The development of high throughput data acquisition and processing methods offers an unprecedented opportunity to overcome the biomass estimation problems at fine levels. This study collected high accuracy terrestrial lidar data at plot, individual plant, leaf group, stem, and individual leaf levels with both platform-mounted and tripod-mounted scanning methods. Based on these data, phenotypic traits were extracted using state-of-the-art algorithms with human refinements in the LiDAR360 software. We used SR, SMR, ANN, and RF methods to evaluate the biomass estimation accuracy and analyzed the important variables at all levels. This study highlighted that (1) the potential of terrestrial lidar data for biomass estimation at fine levels, showing that the accuracy of biomass estimation at fine levels are higher than at plot level; (2) all selected models are suited for biomass estimation, but SR is more transferable and interpretable; (3) height-related variables are the most important and robust phenotypes for predicting maize biomass from terrestrial lidar at all levels, while some 2D and 3D phenotypes, such as leaf area, volume, and 3DPI, also show great potential. These findings demonstrated the great advantages of using lidar data in maize biomass estimation and may contribute to the development of precision agriculture and improved food security.

## Supplementary information

**Additional file 1: Figure S1.** Point cloud examples of three randomly selected profiles with a width of 0.4 m.

## Data Availability

The data and materials used in this study are available from the corresponding author on reasonable request.

## References

[1] Dempewolf H, Bordoni P, Rieseberg LH, Engels JM (2010). Food security: crop species diversity. Science.

[2] Finger R (2011). Food security: close crop yield gap. Nature.

[3] Zhao C (2009). Research and practice of precision agriculture.

[4] Mohamad O, Suhaimi O, Abdullah MZ. The relationships between harvest index, grain yield and biomass in rice. MARDI Res J. 1994;29–34.

[5] Wang C, Nie S, Xi X, Luo S, Sun X (2017). Estimating the biomass of maize with hyperspectral and lidar data. Remote Sens..

[6] Li W, Niu Z, Huang N, Wang C, Gao S, Wu C (2015). Airborne LiDAR technique for estimating biomass components of maize: a case study in Zhangye City, Northwest China. Ecol Indic..

[7] Catchpole WR, Wheeler CJ (1992). Estimating plant biomass: a review of techniques. Aust J Ecol.

[8] Osborne SL, Schepers JS, Francis DD, Schlemmer MR (2002). Use of spectral radiance to estimate in-season biomass and grain yield in nitrogen-and water-stressed corn. Crop Sci.

[9] Liu J, Pattey E, Miller JR, McNairn H, Smith A, Hu B (2010). Estimating crop stresses, aboveground dry biomass and yield of corn using multi-temporal optical data combined with a radiation use efficiency model. Remote Sens Environ.

[10] Chen J, Gu S, Shen M, Tang Y, Matsushita B (2009). Estimating aboveground biomass of grassland having a high canopy cover: an exploratory analysis of in situ hyperspectral data. Int J Remote Sens.

[11] Eitel JU, Magney TS, Vierling LA, Brown TT, Huggins DR (2014). Lidar based biomass and crop nitrogen estimates for rapid, non-destructive assessment of wheat nitrogen status. Field Crop Res..

[12] Gao S, Niu Z, Huang N, Hou X (2013). Estimating the Leaf Area Index, height and biomass of maize using HJ-1 and RADARSAT-2. Int J Appl Earth Obs..

[13] Lin Y (2015). LiDAR: an important tool for next-generation phenotyping technology of high potential for plant phenomics?. Comput Electron Agr..

[14] Guo Q, Wu F, Pang S, Zhao X, Chen L, Liu J, Xue B, Xu G, Li L, Jing H, Chu C (2017). Crop 3D—a LiDAR based platform for 3D high-throughput crop phenotyping. Sci China Life Sci..

[15] Hudak AT, Strand EK, Vierling LA, Byrne JC, Eitel JU, Martinuzzi S, Falkowski MJ (2012). Quantifying aboveground forest carbon pools and fluxes from repeat LiDAR surveys. Remote Sens Environ.

[16] Popescu SC (2007). Estimating biomass of individual pine trees using airborne lidar. Biomass Bioenergy.

[17] Lim K, Treitz P, Wulder M, St-Onge B, Flood M (2003). Lidar remote sensing of forest structure. Prog Phys Geog..

[18] Höfle B (2013). Radiometric correction of terrestrial lidar point cloud data for individual maize plant detection. IEEE Geosci Remote Sens..

[19] Stovall AE, Vorster AG, Anderson RS, Evangelista PH, Shugart HH (2017). Non-destructive aboveground biomass estimation of coniferous trees using terrestrial lidar. Remote Sens Environ.

[20] Momo Takoudjou S, Ploton P, Sonké B, Hackenberg J, Griffon S, De Coligny F, Kamdem NG, Libalah M, Mofack GI, Le Moguédec G, Pélissier R (2018). Using terrestrial laser scanning data to estimate large tropical trees biomass and calibrate allometric models: a comparison with traditional destructive approach. Methods Ecol Evol.

[21] Eitel JU, Magney TS, Vierling LA, Greaves HE, Zheng G (2016). An automated method to quantify crop height and calibrate satellite-derived biomass using hyper temporal lidar. Remote Sens Environ.

[22] Tilly N, Hoffmeister D, Cao Q, Huang S, Lenz-Wiedemann V, Miao Y, Bareth G (2014). Multitemporal crop surface models: accurate plant height measurement and biomass estimation with terrestrial laser scanning in paddy rice. J Appl Remote Sens.

[23] Walter JD, Edwards J, McDonald G, Kuchel H (2019). Estimating biomass and canopy height with lidar for field crop breeding. Front Plant Sci..

[24] Jimenez-Berni JA, Deery DM, Rozas-Larraondo P, Condon AT, Rebetzke GJ, James RA, Bovill WD, Furbank RT, Sirault XR (2018). High throughput determination of plant height, ground cover, and above-ground biomass in wheat with lidar. Front Plant Sci..

[25] Jin S, Su Y, Gao S, Wu F, Hu T, Liu J, Li W, Wang D, Chen S, Jiang Y, Pang S, Guo Q (2018). Deep Learning: individual maize segmentation from terrestrial lidar data using faster R-CNN and regional growth algorithms. Front Plant Sci..

[26] Jin S, Su Y, Gao S, Wu F, Ma Q, Xu K, Hu T, Liu J, Pang S, Guan H, Zhang J, Guo Q (2019). Separating the structural components of maize for field phenotyping using terrestrial lidar data and deep convolutional neural networks. IEEE T Geosci Remote..

[27] Li A, Glenn NF, Olsoy PJ, Mitchell JJ, Shrestha R (2015). Aboveground biomass estimates of sagebrush using terrestrial and airborne LiDAR data in a dryland ecosystem. Agr Forest Meteorol..

[28] Su Y, Wu F, Ao Z, Jin S, Qin F, Liu B, Pang S, Liu L, Guo Q (2019). Evaluating maize phenotype dynamics under drought stress using terrestrial lidar. Plant methods..

[29] Hosoi F, Omasa K (2009). Estimating vertical plant area density profile and growth parameters of a wheat canopy at different growth stages using three-dimensional portable lidar imaging. ISPRS J Photogramm..

[30] Omasa K, Hosoi F, Konishi A (2006). 3D lidar imaging for detecting and understanding plant responses and canopy structure. J Exp Bot.

[31] Jin S, Su Y, Wu F, Pang S, Gao S, Hu T, Liu J, Guo Q (2018). Stem-leaf segmentation and phenotypic trait extraction of individual maize using terrestrial lidar data. IEEE T Geosci Remote..

[32] Yamashita T, Yamashita K, Kamimura R (2007). A stepwise AIC method for variable selection in linear regression. Commun Stat Theory Methods..

[33] Xu K, Su Y, Liu J, Hu T, Jin S, Ma Q, Zhai Q, Wang R, Zhang J, Li Y, Liu H, Guo Q (2019). Estimation of degraded grassland aboveground biomass using machine learning methods from terrestrial laser scanning data. Ecol Indic..

[34] Abrougui K, Gabsi K, Mercatoris B, Khemis C, Amami R, Chehaibi S (2019). Prediction of organic potato yield using tillage systems and soil properties by artificial neural network (ANN) and multiple linear regressions (MLR). Soil Till Res..

[35] Mayilvaganan MK, Naidu KB (2011). ANN and fuzzy logic models for the prediction of groundwater level of a watershed. Int J Comput Sci Eng..

[36] Olden JD, Joy MK, Death RG (2004). An accurate comparison of methods for quantifying variable importance in artificial neural networks using simulated data. Ecol Model.

[37] Breiman L (2001). Random forests. Mach Learn..

[38] Belgiu M, Drăguţ L (2016). Random forest in remote sensing: a review of applications and future directions. ISPRS J Photogramm..

[39] Liaw A, Wiener M (2002). Classification and regression by randomForest. R news..

[40] Wang D, Xin X, Shao Q, Brolly M, Zhu Z, Chen J (2017). Modeling aboveground biomass in Hulunber grassland ecosystem by using unmanned aerial vehicle discrete lidar. Sensors..

[41] García M, Riaño D, Chuvieco E, Danson FM (2010). Estimating biomass carbon stocks for a Mediterranean forest in central Spain using lidar height and intensity data. Remote Sens Environ.

[42] Minh DH, Ndikumana E, Vieilledent G, McKey D, Baghdadi N (2018). Potential value of combining ALOS PALSAR and Landsat-derived tree cover data for forest biomass retrieval in Madagascar. Remote Sens Environ.

[43] Moreira FF, Hearst AA, Cherkauer KA, Rainey KM (2019). Improving the efficiency of soybean breeding with high-throughput canopy phenotyping. Plant Methods..

[44] Tao S, Guo Q, Li L, Xue B, Kelly M, Li W, Xu G, Su Y (2014). Airborne lidar-derived volume metrics for aboveground biomass estimation: a comparative assessment for conifer stands. Agric Forest Meteorol..

[45] Næsset E, Bollandsås OM, Gobakken T, Solberg S, McRoberts RE (2015). The effects of field plot size on model-assisted estimation of aboveground biomass change using multitemporal interferometric SAR and airborne laser scanning data. Remote Sens Environ.

[46] Zhu X, Wang T, Skidmore AK, Darvishzadeh R, Niemann KO, Liu J (2017). Canopy leaf water content estimated using terrestrial lidar. Agric Forest Meteorol..

[47] Zhu X, Wang T, Darvishzadeh R, Skidmore AK, Niemann KO (2015). 3D leaf water content mapping using terrestrial laser scanner backscatter intensity with radiometric correction. ISPRS J Photogramm..

