# Supplementary material for: Non-destructive estimation of field maize biomass using terrestrial lidar: an evaluation from plot level to individual leaf level
Source: Plant Methods. 2020 May 13;16:69. doi: 10.1186/s13007-020-00613-5 (PMC7222476; doi:10.1186/s13007-020-00613-5)
Supplement: Supplementary file 1 — Additional file 1: Figure S1. Point cloud examples of three randomly selected profiles with a width of 0.4 m. [file 13007_2020_613_MOESM1_ESM.docx]

**Additional file 1**

**
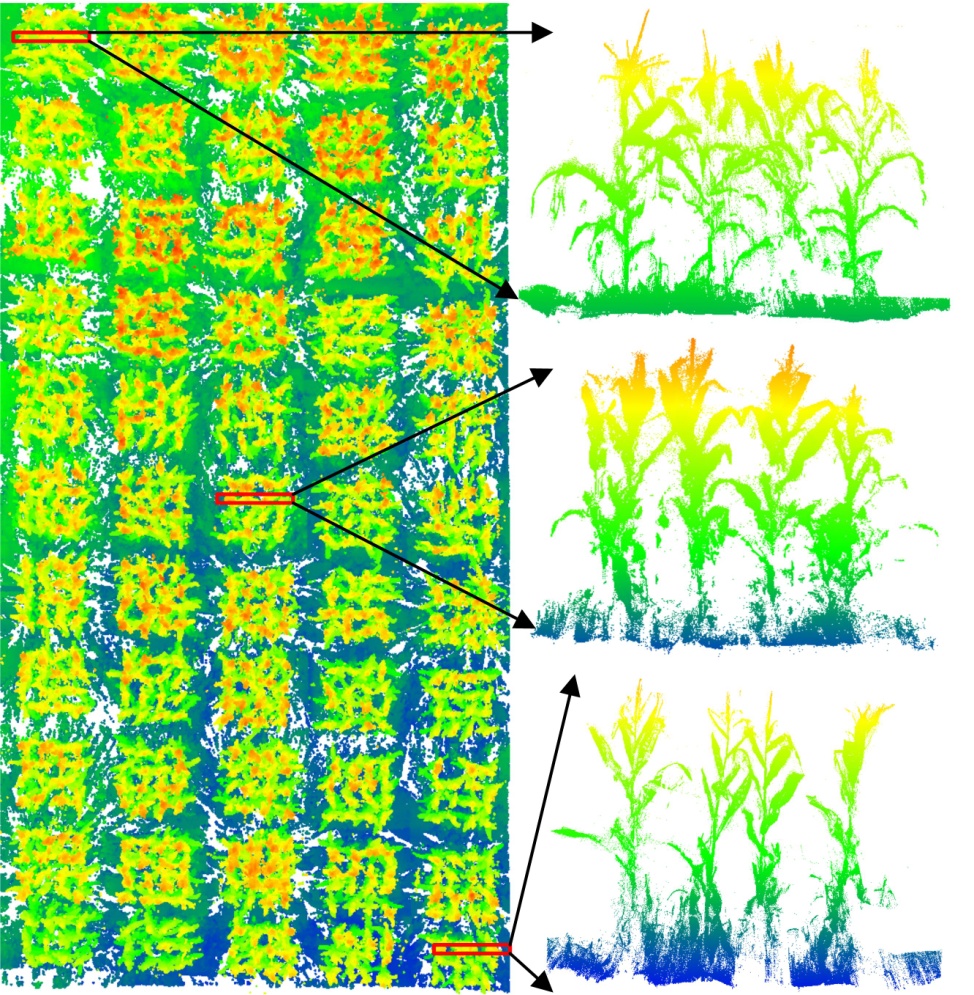
**

**Figure S1.** Point cloud examples of three randomly selected profiles with a width of 0.4 m
